# Investigation on Wax Deposition Reduction Using Natural Plant-Based Additives for Sustainable Energy Production from Penara Oilfield Malaysia Basin

**DOI:** 10.1021/acsomega.2c01333

**Published:** 2022-08-22

**Authors:** Amni Haslinda Alpandi, Hazlina Husin, Syaza Izzaty Jeffri, Akhmal Sidek, Lim Mingyuan

**Affiliations:** † Department of Petroleum Engineering, Universiti Teknologi PETRONAS, Seri Iskandar 32610, Perak, Malaysia; ‡ Department of Petroleum Engineering, School of Chemical and Energy Engineering, Universiti Teknologi Malaysia, Skudai 81310, Johor, Malaysia; § Department of Bioprocess Technology, Faculty of Biotechnology and Biomolecular Sciences, Universiti Putra Malaysia, Serdang 43400, Selangor, Malaysia

## Abstract

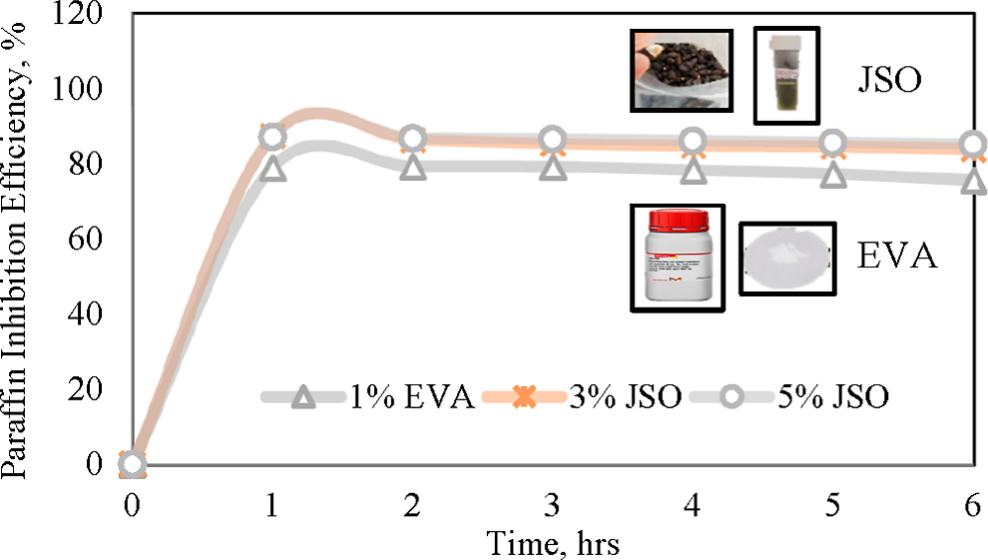

In empowering the United Nations (UN) Sustainable Development Goal, the oil industry is inclined toward organic wax inhibitor applications when combatting the wax deposition issue during crude oil production. This is because synthetic chemical inhibitors are costly and have the potential to create environmental problems when oil spillage or seepage occurs during transportation or operation. This study evaluates the impact of low-cost, natural plant-based inhibitors such as Jatropha seed oil (JSO), crude palm oil, and crude palm kernel oil (CPKO) on paraffin inhibition efficiency (PIE, %) and rheological properties of Malaysian waxy crude oil. By using cold finger equipment and a Fann viscometer, the amount of solid wax deposits, apparent viscosity, plastic viscosity, yield value, and gel strength were determined. Commercialized ethylene-*co*-vinyl acetate and triethanolamine compounds were used for a comparative study. For the wax deposition test, the results revealed that the highest average PIE obtained was 86.30% when 5% JSO was blended with Penara crude oil. Meanwhile, the rheological test proved that 5% JSO and 1% CPKO were acting as highest viscosity-reducing agents at 60 °C below the wax appearance temperature (WAT). The discovery of palm-based and Jatropha-based inhibitors from Malaysia’s palm oil plantation and Malaysian JSO as a wax inhibitor was found to be beneficial for the application of wax deposition and rheological studies in crude oil production with a less harmful environment for sustainable energy production.

## 1 Introduction

Oil and gas exploration in offshore regions which is in ultra-deep, deep, and shallow waters is rapidly increasing due to the gradual depletion of onshore hydrocarbon reserves.^1^ Frigaard et al.^2^ stated that about 20% of the world’s petroleum resources are waxy crude oil. Crude oils have been classified by the American Petroleum Institute (API) into heavy (29 > API), medium (38 > API), and light (API > 38). Due to a very high wax content (>5%), heavy crude oils have the most complex flow behavior.^3^ Wax deposition is the most predominant flow assurance problem caused by the deposition of heavy organic materials present in the oil and leads to the blocking of flow lines.^4^ The organic compounds precipitate, agglomerate, and accumulate from the crude oil stream onto the walls of pipelines and inside process vessels.^5^ According to Thota and Onyeanuna,^6^ the well stream fluid is a multiphase mixture that primarily consists of gaseous hydrocarbon, liquid hydrocarbon (oil), and water. Different problems occur in the pipelines and downhole during the transportation and production of the multiphase fluid, such as scale deposits caused by different inorganic salts, organic depositions (wax and asphaltenes), corrosion damage to the associated equipment and pipelines because of high water cut, and the formation of gas hydrate caused by mixing of hydrocarbons and water. The primary factor accounting for the poor flowability of waxy crude oils is paraffin wax, caused by low temperature.^7^ In crude oil, n-paraffin waxes manage to separate from the oil when the temperature drops below the wax appearance temperature (WAT). The particles will crystallize as the temperature reduces, generate an interlocking network, and entrap the residual liquid fuel in the structures. In the petroleum industry, wax buildup that occurs in light and conventional crude oils is very costly and problematic.^8−10^ According to Ganeeva et al.^11^ and Yang et al.,^12^ the paraffin wax contains at least 15 or more carbon atoms per molecule, which represents saturated and long hydrocarbon chains.

Cold subsea environments affecting the pipe walls in deep water pipelines cause the flowing of crude oil in the pipeline facing a radial thermal gradient. This condition occurs due to the fact that the temperature of crude oil located closer to the pipe wall is lower compared to the center of the pipeline.^13^ The crystallization of wax occurs in the cold region which is located closer to the pipe wall when the crude oil temperature achieves the cloud point. At this point, the equilibrium of solid and liquid phases changes by the crystallization of wax. A concentration gradient appears when the solubility of wax in the crude oil reduces with the presence of thermal energy. The solid wax depositing out from the bulk liquid onto the colder wall of the pipeline is due to the wax concentration gradient. Therefore, wax molecular diffusion appears from the bulk fluid to the wall of the pipeline.^14−16^ Based on Yang et al.,^17^ the wax will develop and deposit on the surface of the pipeline wall when the pipeline wall has a lower temperature than WAT. This scenario explained the molecular diffusion mechanism most simply. Harun et al.,^14^ Leiroz and Azevedo,^15^ and Azevedo and Teixeira^8^ stated that the molecular diffusion effect is very frequent in deep water pipelines. This is because the seabed temperature is near-freezing and causes the submerged pipeline to mimic the near-freeze temperature.

According to Fadairo et al.,^18^ the most efficient method to mitigating the deposition of wax is the chemical method as it deals with the root cause of the wax formation problem. Many researchers such as Popoola et al.,^19^ Atta et al.,^20^ Deshmukh and Bharambe,^21^ and Soni and Bharambe^22^ agreed that the chemical method is able to increase the flowability of waxy crude oil at lower temperatures and prevent the precipitation of wax. They also proved that the chemical method is the most cost-effective and convenient way to solve wax issues. In oilfields, several removal techniques have been applied, such as chemical injection (solvent), thermal method (hot oil or water), and mechanical method (pigging). However, a chemical method such as crystal modifiers and dispersants known as “wax inhibitors” is preferable to inhibit and prevent the deposition of wax.^23^ Even though complete wax inhibition is impossible, it can delay the buildup of wax deposits and reduce the wax deposition rate. Both mechanical and thermal methods are expensive, and this is the major reason behind the extensive use of wax crystal modifiers.^9^ Wax adhesion in the pipeline can be prevented through the injection of chemical additives. These additives can reduce the agglomeration of wax crystals, reduce the pour point, and enhance the flowability of crude oil.^24,25^ Wax that precipitates has an identical chemical structure with the chemical additives which act as wax crystal modifiers. Polymeric compounds represented by typical wax crystal modifiers are constituted of one or more polar portions and hydrocarbon chains (wax-like). By occupying the position of wax molecules on the crystal lattice through hydrocarbon chains, these types of polymeric compounds are able to co-crystallize and co-precipitate with wax. The aggregation and growth of wax crystals can be impeded by a steric hindrance on the crystal and cause a reduction in crude oils to the pour point.^26,27^

Patel et al.,^28^ Soni et al.,^29^ and Hafiz and Khidr^30^ utilized several esters of oleic acid-based polymers as flow improvers by modifying the rheological properties. These potential chemical inhibitors can reduce the yield value, plastic viscosity, and apparent viscosity of Langhnaj crude oil and act as excellent pour point depressants at high concentrations. Besides this, Deka et al.^31^ successfully utilized esters of an oleic acid-based polymer known as tri-triethanolamine to improve flow properties of Indian waxy crude oil. In addition, Yao et al.^32^ successfully used a polyoctadecylacrylate nanocomposite pour point depressant to inhibit wax deposit and improve rheological properties of Changqing waxy crude oil. Recently, a graphene-based nanocomposite has been used by Sharma et al.^33,34^ as a pour point depressant and flow improver for Indian waxy crude oil. Continuous precipitation of wax crystals due to an additional decrease of oil temperature causes the rheology of crude oil to worsen, which leads to huge challenges in pipeline transportation of crude oil.^35−37^ Transportation and rheology of crude oil are among crucial issues to be highlighted in the petroleum industry. According to Ekaputra et al.,^38^ viscosity is defined as a very profound function for the rate of wax deposition and leads to precipitation of wax. In addition, Gudala et al.^39^ stated that viscosity reduction through in situ operation or after production is important to reduce the costs of surface operation. Unfortunately, the current uses of conventional chemical additives such as poly ethylene-*co*-vinyl acetate (EVA), triethanolamine (TEA), and related polymers are not environmentally friendly and very expensive.^40^ Universal paraffin solvents such as carbon disulfide are costly, flammable, and highly toxic when exposed to the environment due to the low flash points.^6^ Therefore, natural plant-based additives which act as wax inhibitors are studied currently to overcome the conventional chemical additives problems. Ragunathan et al.^40^ used edible oil such as crude palm oil (CPO) and crude palm kernel oil (CPKO). Fadairo et al.^18^ used rubber seed oil (RSO) and castor seed oil (CSO) in their studies. Meanwhile, Akinyemi et al.^41^ used RSO, CSO, and Jatropha seed oil (JSO) to mitigate wax deposition in Nigerian waxy crude oil. Further research was conducted by Akinyemi et al.^42^ on the usage of plant seed oil as a wax inhibitor by employing CSO blend with JSO. Other than this, Deka et al.^43^ successfully utilized two polymeric compounds as pour point depressants for waxy crude oil, which were naturally obtained from vegetable oil fatty acids, known as poly (*n*-dodecyl linoleate-*co*-succinic anhydride) and poly (*n*-dodecyl ricinoleate-*co*-succinic anhydride).

Based on the study conducted by Ragunathan et al.,^40^ a positive effect was found on the WAT and paraffin inhibition efficiency (PIE) when CPO and CPKO with 0.1, 1, and 10% concentration have been employed to Mt Mckyle Arab heavy crude oil. However, this type of crude oil is less waxy with a low WAT value, which is 14.89 °C only. Besides this, Ragunathan et al.^44^ also conducted the rheological behavior of crude oil in the presence of CPO and CPKO. However, the crude oil used from the Chenor oil field in the Malaysia basin is less waxy with a low WAT value, which is 35.00 °C only. Therefore, the impact of CPO and CPKO in mitigating the deposition of waxier crude oil and their rheological behavior is still uncertain. Moreover, Akinyemi et al.^41^ successfully used JSO from the Nigeria region to mitigate wax deposition of Nigerian waxy crude oil. Jayagobi et al.^45^ found that JSO from the Malaysia region has potential as a wax inhibitor due to a high content of oleic acid with 44.91% compared to Nigerian waxy crude oil (43.11%). This non-edible oil is useful in mitigating wax deposition in aging reservoirs such as Malay basin. However, the effect of Malaysian JSO upon wax inhibition of waxy crude oil is still uncertain. This work is aimed to investigate the impacts of natural plant-based additives such as CPO, CPKO, and Malaysian JSO on PIE and rheological properties of waxy crude oil from Penara oilfield Malaysia basin, which is waxier compared to Mt Mckyle Arab heavy crude oil and Chenor crude oil at different concentrations, temperatures, and shear rates. In addition, the impact of TEA and EVA on the crude oil samples were also investigated for comparative purposes. The concentration of additives also varies from 1 to 5% to ensure the optimum concentration and ability to inhibit the deposition of wax effectively. Once the paraffin wax deposition is successfully mitigated by using natural plant-based additives, this condition will contribute to high sustainable energy production.

## 2 Materials and Methods

### 2.1 Materials

CPO and CPKO were obtained from Unitata company and United Plantation, Malaysian JSO was obtained from Universiti Putra Malaysia, and TEA and EVA used were products of Sigma Aldrich. The crude oil samples were obtained from Penara oilfield, Malaysia basin.

### 2.2 Sample Preparation

**Figure 1:**
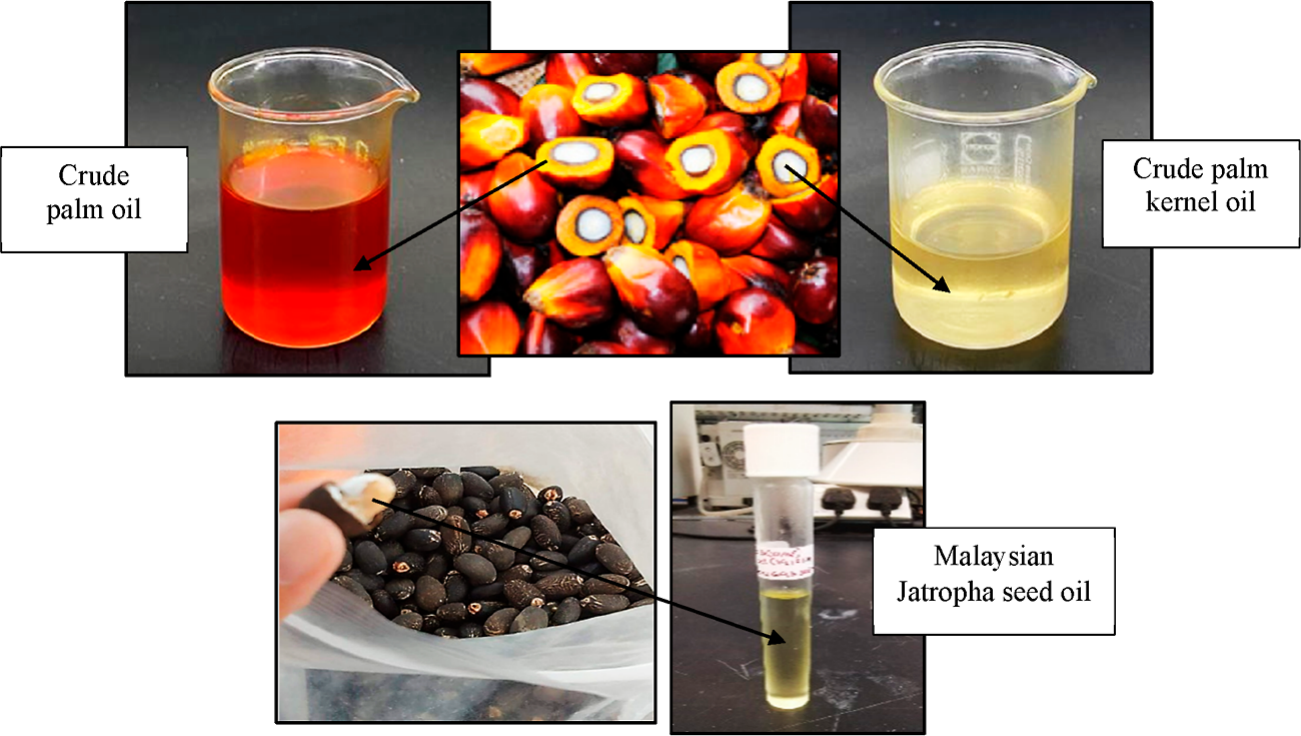
CPO, CPKO, and Malaysian JSO samples.

The crude oil sample was preheated in an oven for about 1 h until the temperature reached was 60 °C to remove any unwanted history that exists in the sample and to ensure the condition that crude oil easily flows. Reconditioning the sample is needed to ensure that all pre-crystallized wax re-dissolved into the oil. Any shear and thermal history were removed throughout this process, and a homogeneous sample was produced for testing. Then, additives such as CPO were added at different concentrations (1, 3, and 5%) into three different crude oil samples, and the mixtures were stirred until it was homogeneous. A similar procedure for sample preparation was repeated by using other types of wax inhibitors, which are CPKO, Malaysian JSO, TEA and EVA. Figure 1 shows CPO, CPKO, and Malaysian JSO samples used in this study.

### 2.3 Characterization of Crude Oil Samples

The chemical properties of crude oil samples such as density, specific gravity (SG), API gravity, and wax content are shown in Table 1.^46,47^ Penara crude oil is waxy and has complex flow behavior due to a very high wax content with 18.00 wt %.

**Table 1:** Chemical Properties of Penara Crude Oil

chemical properties	unit	value
density	g/cm^3^	0.9165
SG		0.917
API gravity	API	22.80
wax content	wt %	18.00

Characterization of the crude oil was conducted in the lab using standard analytical techniques and experimental methods. The WAT was determined by using a micro-differential scanning calorimetry (μDSC) 7 Evo IV model supplied by SETARAM Instrumentation, United Kingdom. The μDSC system consists of a 3D Calvet thermal sensor, reference cells, and a closed sample for better accuracy and precision, particularly for measurements at a low cooling rate. The chamber was flushed by using nitrogen gas to eliminate any contaminant and producing an inert atmosphere around the cell of the sample. With 10 min holding time between sequences, deionized water was used for a calibration from 20 to −20 °C (cooling) and back to 20 °C (heating) to ensure accurate and precise measurements. The crude oil sample used was initially liquified during sample preparation. About 0.05 mL of the crude oil sample was transported into a hastelloy C276 closed batch cell. For a reference cell, another identical hastelloy C276 closed batch cell (empty) was utilized. Without shear, the measurement of WAT was performed under static cooling from 80 to 0 °C at a cooling rate of 0.5 °C/min for the crude oil sample. To ensure a better baseline without wax crystals before the process of wax crystallization started, selection of a high starting temperature is needed. After a heating phase up to 80 °C, the samples were cooled down from 80 to 0 °C at 0.5 °C/min. The onset temperature of the first peak has been considered to calculate the WAT, which is at the intersection of the baseline and the tangent at the first peak inflection point.

Meanwhile, the pour point temperature was determined by using a Pour Point Tester PT 45150 model supplied by PSL Systemtechnik, Germany. It is the equipment used to supply heating and cooling in a cycle mode to the crude oil sample, and then the pour point value was measured by software connected to it. First, the fluid level inside the cooling system is checked. Then, the main unit is switched on to operate the pour point tester. The sample cup holder, temperature sensor, and sample cup of the equipment are cleaned up before use. After that, the crude oil sample is filled up into the cup until the amount reaches the marking ring. The sample cup filled with crude oil is placed into the cup holder and rotated until it is locked. Next, the temperature sensor is lowered to the telescopic tube and the sample. For the software part, the WinPPT software was started, and the water supply was opened by pressing “Open Valve” from the software. The button “Configuration” was pressed, and the input of field “Name” was entered. Then, “Temperature one” was entered as the starting temperature, “Temperature two” was entered as the end temperature, and “no. of cycles” was added for measurements. For the expected pour point higher than −33 °C, the starting temperature (Temperature 1) selected was at least 9 °C above the expected pour point or 45 °C but not higher than 70 °C. Last, the button “Run” was clicked to start the measurement of the pour point sample.

Other than that, carbon number distribution of Penara crude oil was determined using gas chromatography mass spectrometry (GCMS) equipment with Agilent Technologies model 7820A and the capillary column of DB-5MS. All the available components in the crude oil were illustrated in a spectrum result. About 2 mL of Penara waxy crude oil was diluted with 4 mL of hexane before being injected into the gas chromatograph. The procedure of the experiment was commenced by setting the temperature to 120 °C, which was held for 3 min until it reached the maximum temperature of 270 °C, which was held for 40 min. The temperature increasing rate was set at 10 °C/min. At the temperature of 300 °C, splitless injection was carried out with a constant flow rate, which is 0.8 mL/min. The mass spectrometry transfer line was set at 300 °C, and the ion source was kept at 230 °C. The peaks were identified by measuring the retention time of the samples and comparing the same with authentic standards analyzed under the same conditions. The carbon number of all components in Penara waxy crude oil was determined by analyzing the GCMS spectrum.

### 2.4 PIE Measurement

**Figure 2:**
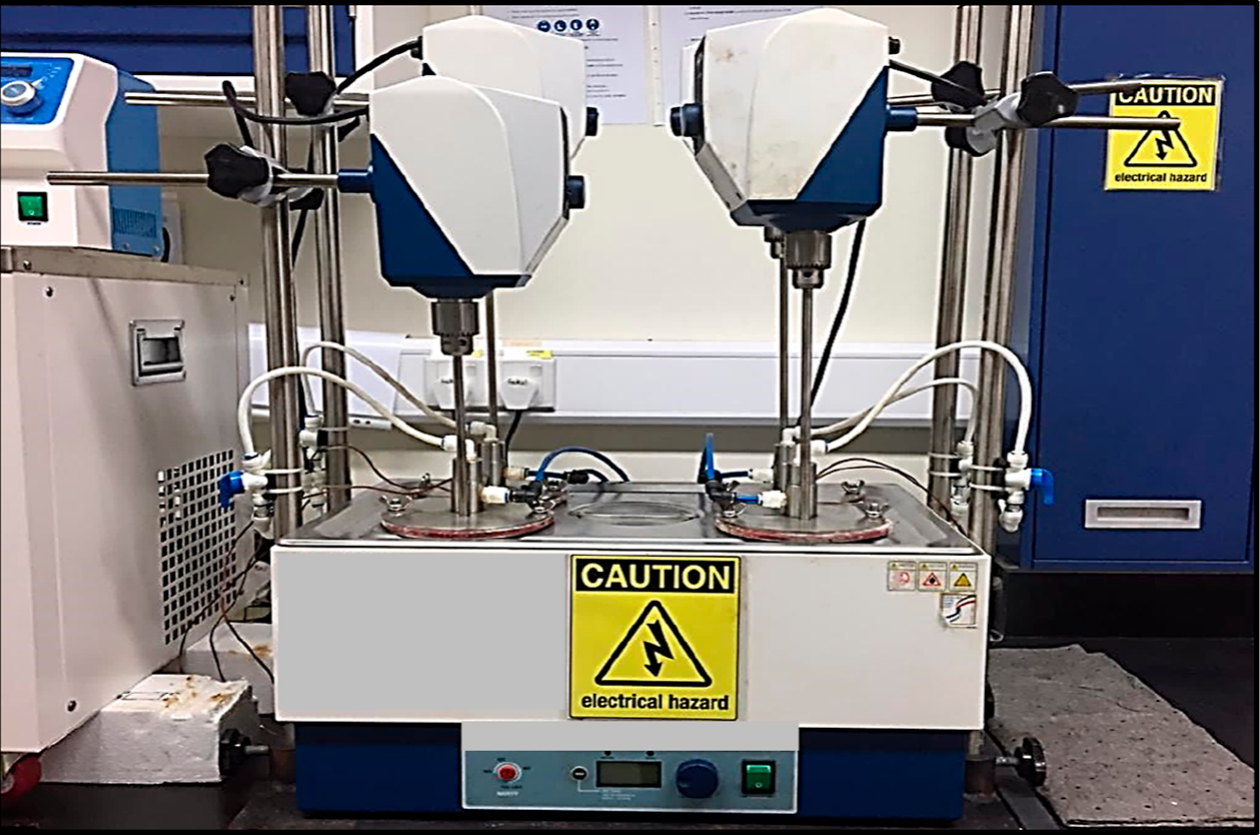
Cold finger system utilized in this study.

A cold finger is the most suitable equipment for wax deposition tests of crude oil samples in a time-friendly manner and lower-cost operation. The cold finger represents the subsea condition where the colder pipe wall is in contact with the warm oil, which finally generates a mechanism of molecular diffusion for deposition of wax.^48^ The measurements of PIE of natural plant-based additives and synthetic chemical wax inhibitors were conducted throughout this research by utilizing a cold finger system under static conditions. Figure 2 shows the cold finger system that has been set up for wax deposition investigation.

At the beginning of this experiment, the crude oil sample without the wax inhibitor was preheated to 60 °C using an oven. Then, the additive was added to the crude oil sample, and the mixture was stirred using a magnetic stirrer until homogeneity. During the utilization of the cold finger, first, 150 mL of the crude oil sample without the inhibitor was poured into the oil tank. The cold finger probe was set at 45 °C, and the bulk point temperature was maintained at 60 °C to produce a temperature gradient of 15 °C. The cold finger probe was removed along with the lid from the apparatus at an interval of 1 h; then, the mass of deposited wax was weighted using a mass balance.

The experiment was run for 6 h to measure wax deposited at various aging times. The experiment was repeated by employing the natural plant-based additives at the concentrations of 1, 3, and 5%, followed by synthetic chemical wax inhibitors at the concentration of 1%. Three oil tanks of the cold finger system are used concurrently for each run of the experiment. After the mass of deposited wax is obtained for each sample, the average amounts of deposited wax are used to calculate the PIE. Equation 1 shows the parameter used to calculate the PIE of each chemical additive
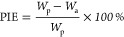
1where *W*_a_ is the mass of the deposited paraffin wax in the presence of the chemical additive and *W*_p_ is the mass of deposited paraffin wax in the absence of an inhibitor.

### 2.5 Rheology Measurement

The rheological testing of crude oil samples with and without chemical additives was conducted using Fann viscometer model 35 manufactured by Fann Instrument Company from Houston, Texas, USA. The crude oil was heated up to 60 °C using an oven. Then, the additives such as CPO, CPKO, Malaysian JSO, TEA, and EVA were added into the crude oil sample, and the mixture was stirred until homogeneity using a magnetic stirrer. The viscosity study was performed using different concentrations of additives (1, 3, and 5%) at different temperatures, which are at 60, 70, and 80 °C for each sample. The rheological measurement was conducted with six different shear rates of the viscometer starting from 600, 300, 200, 100, 6, and 3 rpm. Each of the readings of shear rate was taken when the pointer from the viscometer’s dial was steady.

First, a recently agitated crude oil sample of about 175 mL was placed in the thermo cup. Then, the upper housing of the viscometer was tilted back. The cup was located under the sleeve, and the pins on the bottom of the cup fit into the holes in the base plate. After that, the upper housing was lowered to its normal position, and the knurled knob was turned between the rear support posts to raise or lower the rotor sleeve until it was immersed in the sample to the scribed line. The sample was stirred for about 5 s at 600 rpm, and then the shear rate desired for the best was selected. We waited until the dial reading stabilized (the time depends on the sample’s characteristics). Finally, the dial reading and shear rate were recorded.

The experiment was repeated by adding additives into crude oil samples at different concentrations and temperatures. Based on the readings obtained, the apparent viscosity, plastic viscosity, yield value, and gel strength can be calculated by using standard formulas as stated in eqs 2, 3, 4, and 5, respectively. The measurements were repeated twice, and the results acquired were averaged to ensure the accuracy and reliability of the results^49,50^
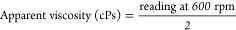
2
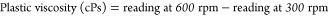
3
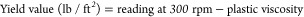
4
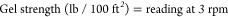
5

## 3 Results and Discussion

### 3.1 Characteristics of Crude Oil

The Penara crude oil with waxing problems used in this study is from one of the oil field reservoirs in Malaysia. All the characterization of crude oil was conducted in the lab following the standard analytical methods. The physicochemical properties of the Penara crude oil sample including its WAT and pour point obtained are shown in Table 2.

**Table 2:** Physicochemical Properties of Penara Crude Oil

chemical properties	unit	value
WAT/cloud point	°C	72.24
pour point	°C	59.25

**Figure 3:**
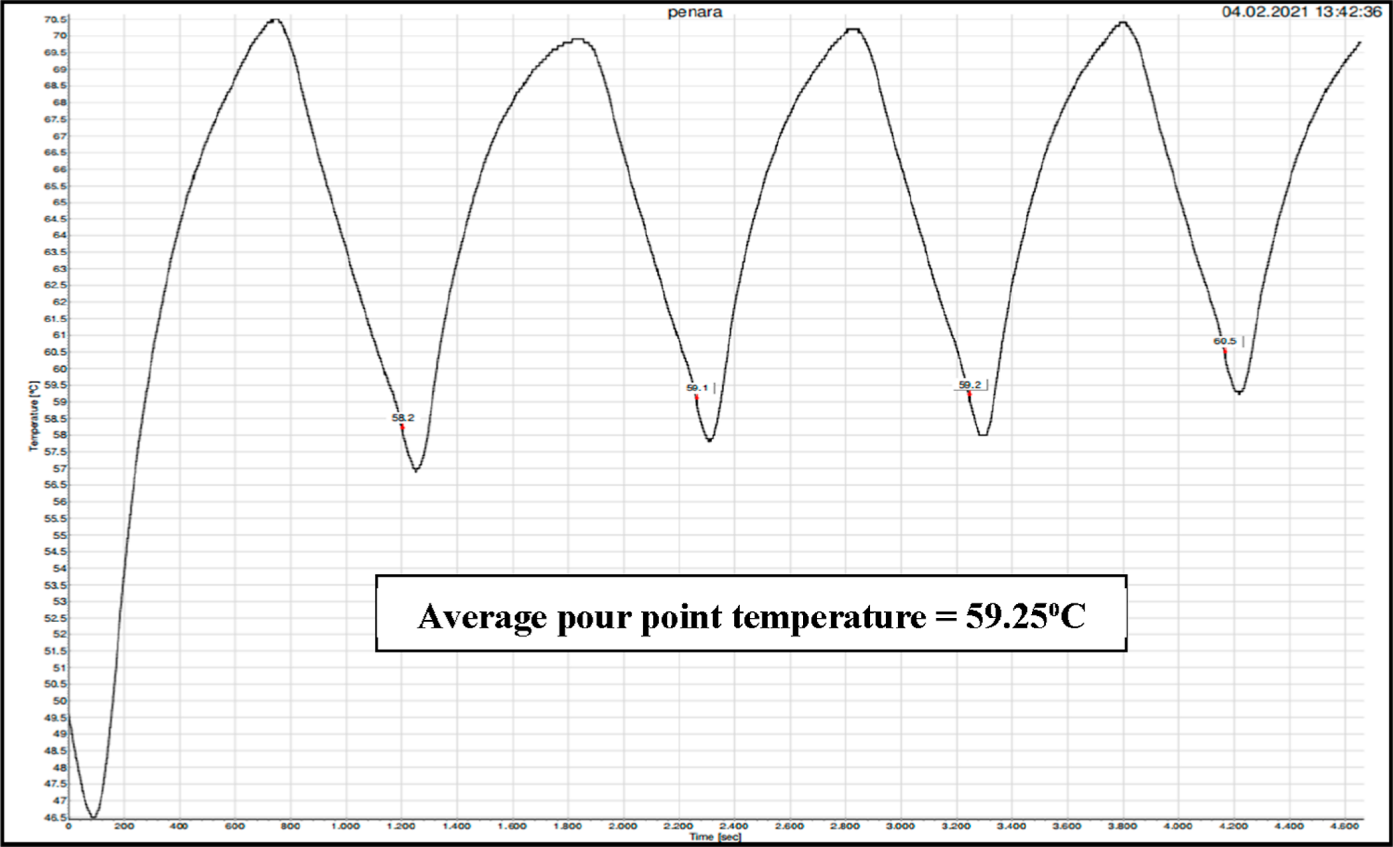
Pour point temperature of Penara crude oil using pour point tester measurements.

The WAT value of the crude oil sample was obtained using μDSC measurements. The WAT value was determined from the graph of heat flow against temperature. From the graph, one exothermic peak was recorded for the Penara crude oil sample corresponding to the crystallization of waxes. The temperature at onset is equal to the WAT value, which is 72.24 °C. The pour point temperature is determined to ensure that the crude oil starts to immobilize and does not flow smoothly as it has gone through the cooling phase. Figure 3 shows the trend of pour point temperature obtained from pour point tester measurements. Based on Figure 3, there are several temperatures obtained to confirm the pour point value of the crude oil. Hence, the average pour point temperature was calculated for Penara crude oil, which is 59.25 °C.

**Figure 4:**
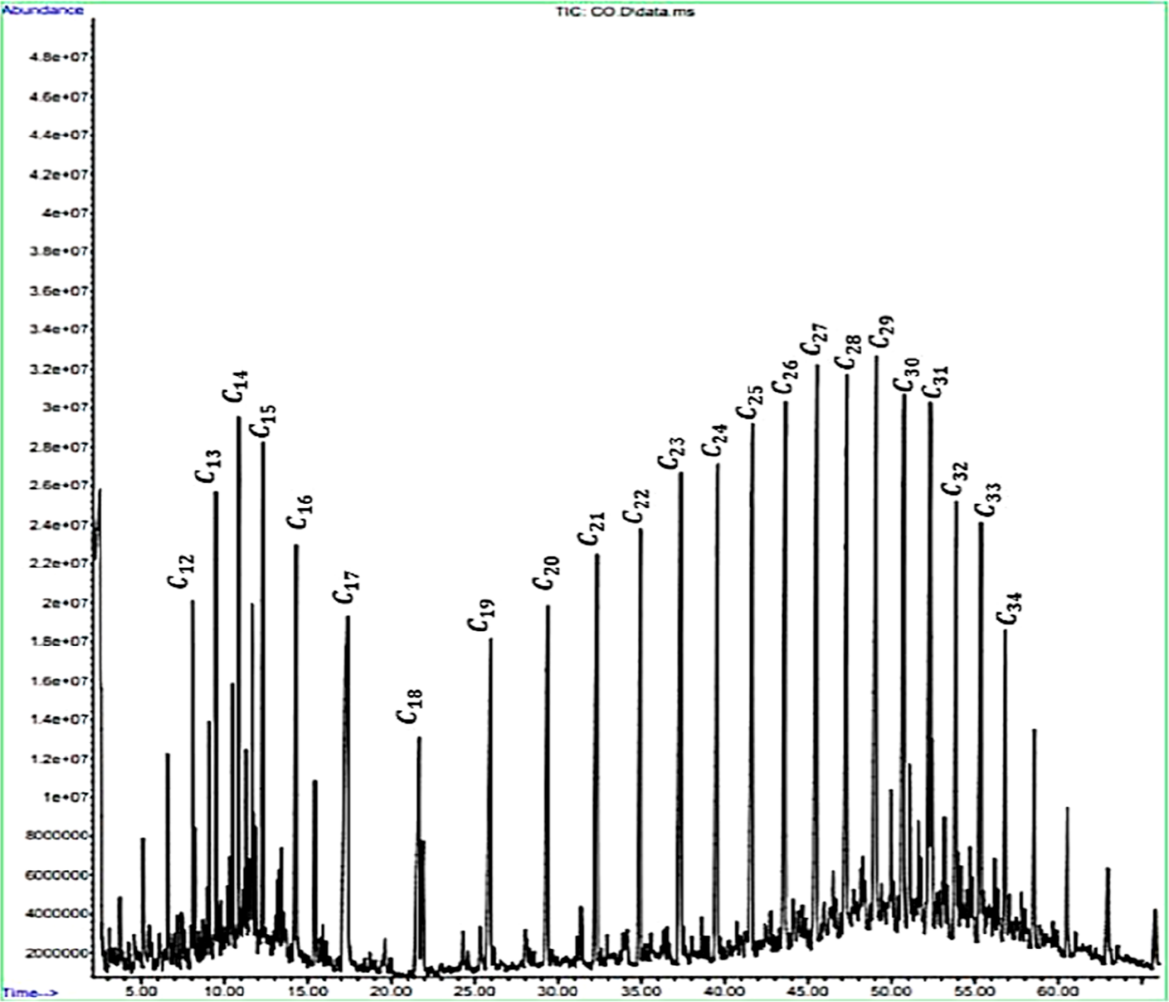
Chromatogram of Penara crude oil.

Hydrocarbon number distribution of Penara crude oil was conducted using GCMS. This test was conducted to analyze the distribution of paraffinic composition hydrocarbon number in the sample. The several peaks in the chromatogram results obtained from GCMS measurements reflect the type of component present in the compound. Figure 4 shows the chromatogram of Penara crude oil used in this study.

Figure 4 displays the chromatogram of Penara crude oil with the *X*-axis representing the retention time and the *Y*-axis representing the abundance of the component. The trend of hydrocarbon number distribution can be clearly seen as the retention time increases. It is spotted that the heavier components are found as the trend moves to the right side, while the lighter components are most abundantly found on the left side of the chromatogram. Based on the analysis, the hydrocarbon number distribution shown is in the range of C_12_ to C_34_. The molecule presented in either straight or branched hydrocarbon chains and might contain some aromatic or cyclic hydrocarbons.

Thota and Onyeanuna^6^ stated that naphthenic hydrocarbons are found mainly in petroleum crudes at C_30_ to C_60_, while paraffin hydrocarbons are found in the range of C_18_ to C_36_, which are much lower numbers of carbon compared to naphthenic hydrocarbons. Due to the high range of hydrocarbon numbers, the Penara crude oil might consist of paraffin wax as wax mainly formed crystalline when it contains normal paraffin with 16 or more carbon atoms (≥C_16_). Therefore, this result confirms that the type of crude oil and the molecular composition of the molecules in the crude oil are several factors affecting the severity of the wax deposition issue.

### 3.2 Effect on the Wax Deposited Amount

**Figure 5:**
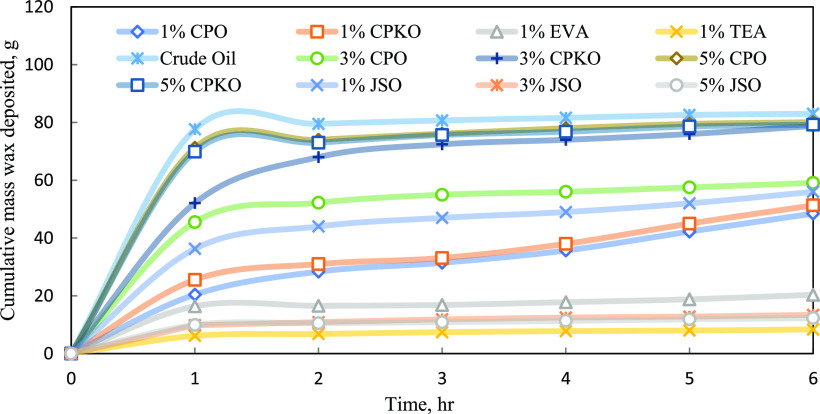
Cumulative mass of wax deposited against the deposition time of crude oil with and without chemical inhibitors.

Natural plant-based additives are tested with different concentrations to investigate their effect on wax inhibition. Based on the experiment conducted, the cumulative mass of wax deposits of crude oil with and without inhibitors is plotted against the deposition time as shown in Figure 5. The measurements are obtained on 1 h basis up to 6 h. The crude oil plot in Figure 5 with inhibitors is included to monitor the implication of cumulative mass of wax deposit by the addition of 1, 3, and 5% of CPO, CPKO, and JSO blends in Penara crude oil. Besides natural plant-based additives, others synthetic chemical inhibitors such as TEA and EVA with 1% concentration have been used to test their efficiency, and the results are needed for a comparative study.

Based on Figure 5, the graph shows that the cumulative mass of wax deposited by Penara crude oil increases with the deposition time. It can be seen that Penara crude oil without the presence of the chemical inhibitor shows the highest amount of wax deposited from the beginning till the end of 6 h deposition time with 83.02 g. However, the trend indicates a significant difference when the chemical inhibitors with different concentrations were added to the base crude oil. The growth of wax deposit mainly depends on inhibitor concentration, and this factor plays an important role in controlling the growth of wax deposit.^51,52^

The addition of 1, 3, and 5% of CPO, CPKO, and JSO additives in base crude oil shows the decreasing amount of wax deposited. However, at 6 h aging, the crude oil sample blend with 5% of JSO obtained the lowest amount of deposited wax, which is 12.30 g, followed by 3% JSO with 13.41 g and 1% CPO with 48.47 g of deposited wax. This result proved that the presence of natural plant-based additives causes the separation of wax molecules from each other and makes interactions among molecules less favorable. The reduction in wax deposition proves that natural plant-based additives can prevent the wax crystal network from interlocking by creating a barrier and then impeding wax deposition.^40^ Hence, the amount of wax deposit at the end of aging will be less, and this shows that natural plant-based additives managed to inhibit a waxy crude oil.

Based on the observation from Figure 5, the amount of wax deposited in the Penara crude oil blend with 1% concentration of CPO, CPKO, JSO, TEA, and EVA additives at 6 h deposition time is 48.47, 51.33, 56.00, 8.38, and 20.35 g, respectively, compared to the base crude oil with 83.02 g. CPO, CPKO, and JSO with 3% concentration reduce the amount of wax deposited from base crude oil to 59.09, 78.78, and 13.41 g at 6 h deposition time. Similarly, at 5% concentration, CPO, CPKO, and JSO are also able to reduce the amount of wax deposited at 6 h deposition time with 80.11, 79.22, and 12.30 g, respectively. All the chemical inhibitors were able to lessen the wax deposit amount at all concentrations tested due to the strong intermolecular forces such as the van der Waals interaction between molecules, the inhibitor, and crystal wax in crude oil.^53,54^ This reflects that natural plant-based additives such as CPO, CPKO, and JSO can perform efficiently as synthetic chemical inhibitors in mitigating wax deposition.

**Figure 6:**
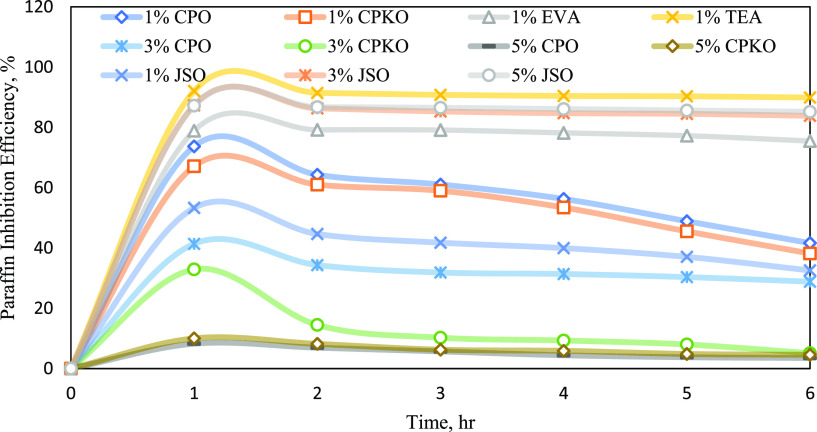
PIE against deposition time.

**Figure 7:**
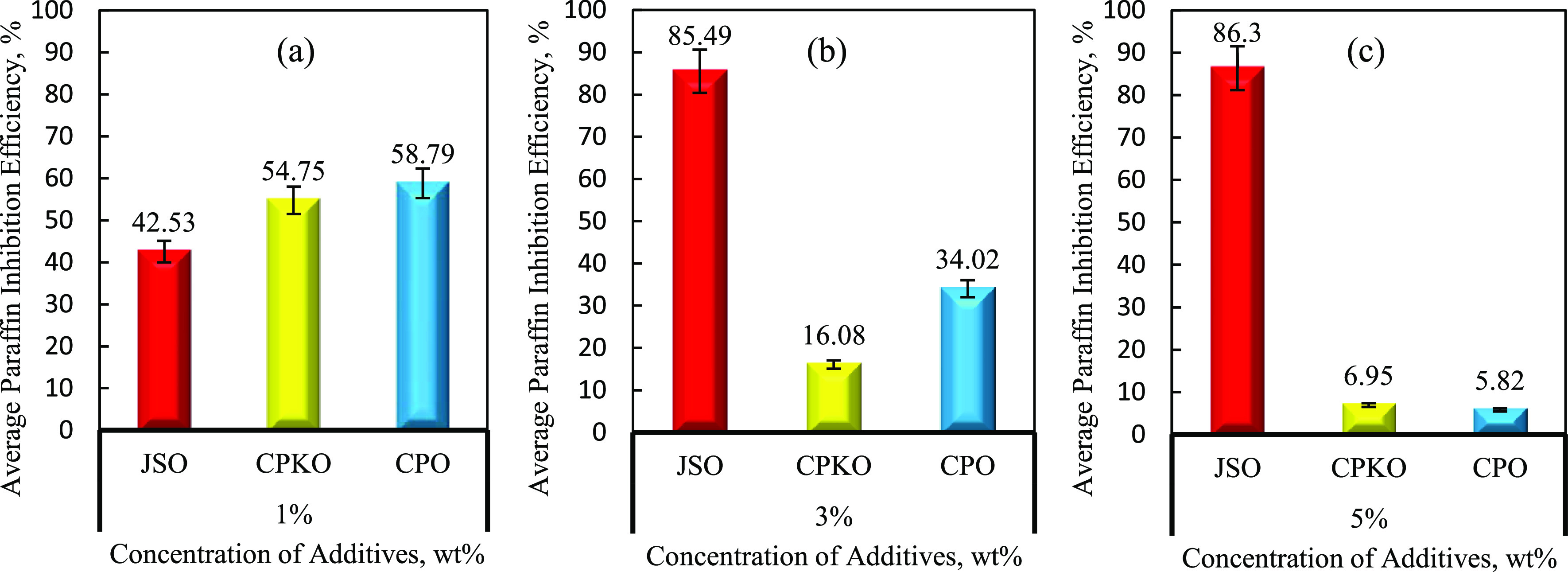
Average PIE against the concentration of additives. (a) 1% concentration, (b) 3% concentration, and (c) 5% concentration. Data presented are with 6% error bar.

To gain a better understanding of the efficiency of natural plant-based additives as a wax inhibitor, the graph on PIE against deposition time and the bar chart on average PIE against the concentration of additives were plotted, as shown in Figures 6 and 7, respectively.

According to Figure 6, the graph demonstrates the PIE against deposition time for various types of chemical additives with different concentrations. Referring to the results obtained, 5% JSO has the highest PIE at the end of 6 h deposition time, followed by 3% JSO, 1% CPO, 1% CPKO, 1% JSO, 3% CPO, 3% CPKO, 5% CPKO, and finally 5% CPO.

It can be seen that 5% JSO, 3% JSO, 1% CPO, 1% CPKO, 1% JSO, and 3% CPO reached a plateau level of PIE at approximately 1.3 h of aging time; meanwhile, 3% CPKO, 5% CKPO, and 5% CPO reached a plateau level of PIE at approximately 1 h of aging time. After that, the performance of natural plant-based inhibition efficiency was independent of aging time. This trend demonstrates that natural plant-based additives perform best as wax chemical inhibitors with a threshold concentration.^40^ The order of natural plant-based additive performances is summarized as per the following sequence in descending order:

5% JSO > 3% JSO > 1% CPO > 1% CPKO > 1% JSO > 3% CPO > 3% CPKO > 5% CPKO > 5% CPO.

Since the natural plant-based additives show great performance in mitigating wax deposition, paraffin wax inhibitors are further compared with TEA and EVA additives at 1% concentration. The outcome reveals that 3 and 5% JSO are more efficient as compared to 1% EVA but less efficient than 1% TEA. TEA is a cationic surfactant that has surface active agents where the surface activity depends on carbon side chain length and the nature of the quaternized amine.^55^ However, with 1% concentration TEA gives high performance in mitigating wax deposition due to the properties, acting as potential sites for wax agglomeration. Besides this, EVA contains multiple active oxygen atoms and comprises the methylene group which displays a strong van der Waals interaction between hydrogen atoms in the crude oil. These properties contribute to declination in wax deposition due to the reduction of the wax gel strength and increased solubility.^27,56^ EVA can improve the flow behavior of waxy oils by modifying the morphology of wax crystals from long needle-like particles into fine wax crystal particles.^57^

However, TEA and EVA are expensive and give a negative impact on the environment due to their chemical properties, which are unable to decompose as easily as natural plant-based additives. Both synthetic chemical inhibitors possess toxicity problems when they are exposed to nature, which happens during transportation or operation or is caused by spillage and seepage. Therefore, this study proved that natural plant-based additives such as CPO, CPKO, and Malaysian JSO can be one of the alternatives in mitigating wax deposition with a more environmentally friendly effect and much cheaper.^40,45^ To further investigate the implication of concentration factor, average PIE against concentration of natural plant-based additives was studied, as shown in Figure 7. Averaging the weight of wax deposited through the 6 h duration is performed to indicate the pro-rate basis within a contained condition or incubated condition. This means that there is only one PIE % value to represent the performance of additive at one concentration.

Figure 7 shows the average PIE against the concentration of additives when (a) at 1% concentration, (b) at 3% concentration, and (c) at 5% concentration. It is clearly seen that the average PIE of JSO (red bar) increases from 42.53% to 86.30% with increasing concentration of additive from 1 to 5%. However, the average PIE of CPKO (yellow bar) continuously decreases from 54.75% at 1% concentration to 6.95% at 5% concentration. CPO (blue bar) also shows the same pattern as CPKO with decreasing average of PIE from 1% to 5% concentration with 58.79% and drop to 5.82% as shown in Figure 7a–c. The trend of the bar chart in Figure 7 shows that JSO gives the highest average PIE compared to CPO and CPKO at 5% concentration with 86.30% as shown in Figure 7c, followed by 3% JSO with 85.49% of average PIE as shown in Figure 7b.

The average PIE increases with increasing concentration of JSO. These results indicate that non-edible oils such as Malaysian JSO used in this study perform better as natural plant-based wax inhibitors compared to CPO and CPKO in mitigating paraffin wax deposition of waxy crude oil from Malaysia basin. According to Jayagobi et al.,^45^ Malaysian JSO has a high content of oleic acid with 44.91%, which is highly capable as a wax inhibitor in decreasing wax deposition. Malaysian JSO has a decrease in ester fatty acid groups due to the presence of C=O stretching carboxyl. The oleic acid content in Malaysian JSO is higher compared to others natural plant-based inhibitors. Table 3 shows the comparison of plant-based wax inhibitors between this study and published findings.

**Table 3:** Comparison of Plant-Based Wax Inhibitors between This Study and Published Findings

reference	plant-based wax inhibitor	type of oil (non-edible/edible)	oleic acid content, %
this study	Malaysian JSO	non-edible	44.91
Ragunathan et al.^40^	CPO	edible	44.10
Ragunathan et al.^40^	CPKO	edible	15.30
Akinyemi et al.^41^	Nigerian JSO	non-edible	43.11
Emil et al.^58^	Indonesian JSO	non-edible	42.40

**Figure 8:**
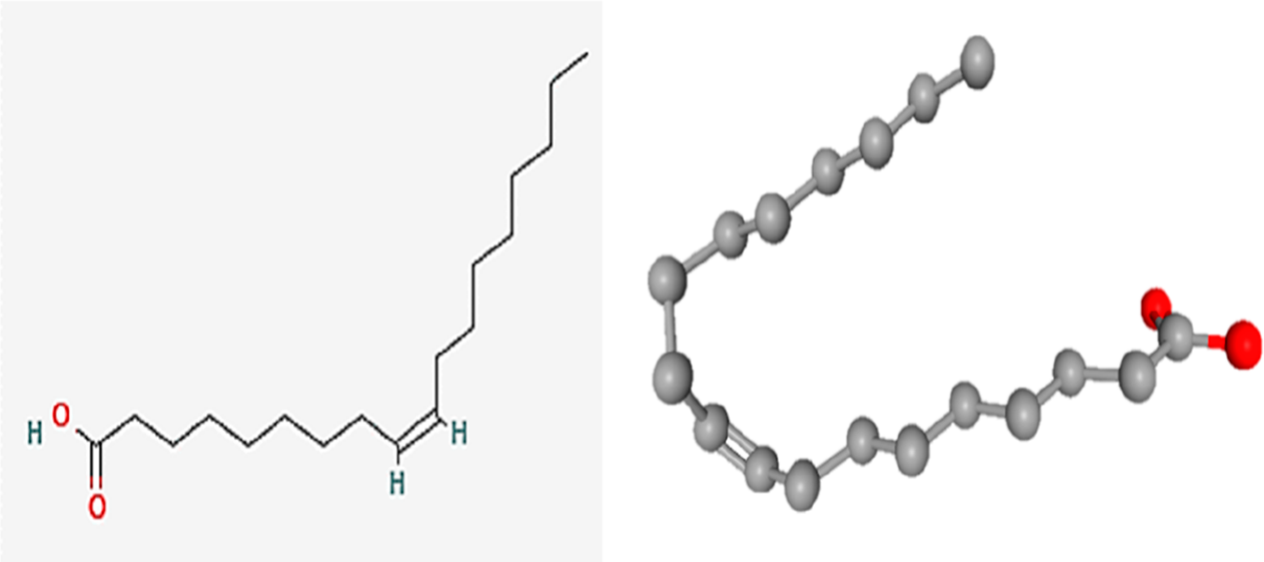
Chemical structure of oleic acid in Malaysian JSO.

Oleic acid is the main active component in Malaysian JSO which is involved in the interaction with crude oil containing active hydrocarbons. These interactions between molecules lead to wax deposition inhibition. The molecules of monosaturation present in the JSO are capable of attaching to the larger molecules of paraffin in solution, and this behavior is able to separate them from generating wax aggregation and build up wax deposition. Figure 8 shows the chemical structure of oleic acid found in Malaysian JSO. According to Alpandi et al.,^59^ JSO was found as the most suitable wax inhibitor because this type of natural plant-based additive is cheap, non-toxic to the nature, and non-edible. In addition, JSO is accepted by the industry, and its application as a flow improver is able to cover a wide range of crude oil fields.^41,42^ Jayagobi et al.^45^ stated that Malaysian JSO is efficient in inhibiting one of the serious flow assurance problems, especially in aging reservoirs such as the Malay basin. Therefore, this study proved that Malaysian JSO is able to inhibit wax deposition of Penara waxy crude oil from Malaysia basin efficiently at 5% concentration.

CPO and CPKO are edible oils and known as palm oil inhibitors. Based on Figure 7a, it can be observed that CPO has a high average PIE with 58.79% compared to CPKO with 54.75% at 1% concentration. CPKO was able to inhibit paraffin wax in Penara waxy crude oil. However, CPO shows better performance compared to CPKO due to the higher amount of oleic acid present in CPO. In such edible oils, there is a presence of fatty acid components with different compositions formed naturally. The most abundant fatty acid components in these palm oil inhibitors used in this study are oleic acid and palmitic acid. The difference between each acid component is that oleic acid is an 18-carbon unsaturated fatty acid; meanwhile, palmitic acid consists of a 16-carbon saturated fatty acid. Based on this study, unsaturated fatty acids such as oleic acid are the main factor that contributes to the efficiency of the palm oil inhibitors in mitigating wax deposition. The oleic acid able to bind with larger paraffin molecules in the solution thereby helps to sequester them from wax coagulation. Hence, the reason behind the outstanding performance of CPO is the highest oleic acid content with 44.10% compared to CPKO with 15.30% oleic acid content only.^40^

Furthermore, the trend of the bar chart in Figure 7a–c illustrates that the average PIE for both CPO and CPKO is seen to be decreased when the concentration increases from 1 to 5%. This is because the molecules from the additives which act as nucleating sites for the paraffin wax will undergo crystallization. Thus, this study answered the question from Ragunathan et al.,^40^ in which the optimum concentration of palm oil-based inhibitors to mitigate wax accumulation is proven at 1% CPO and CPKO compared to other concentrations tested as this concentration shows the greatest efficiency in mitigating paraffin wax deposition.

Ragunathan et al.^40^ suggested utilizing these palm oil inhibitors under different types of crude oil and preferably waxier. This further research is needed to ensure the efficiency of CPO and CPKO as a wax inhibitor. Therefore, this study was conducted using Malaysia waxy crude oil from the Penara well which has a high WAT, a high pour point, and a high wax content, which are 72.24 °C, 59.25 °C, and 18.00 wt %, respectively. Compared to Mt Oversea Mckyle crude oil, this type of crude oil has a low value of WAT, pour point, and wax content with 14.89 °C, −18.00 °C, and 7.51 wt %, respectively.^40^

According to Beiny et al.,^60^ various research studies stated that it is sufficient for gelling of a virgin waxy crude oil to be induced with approximately 2 wt % of paraffin wax. The amount and thickness of wax that will be deposited during the transportation of crude oil are determined by the wax content existing in the crude oil. The higher the wax content, the higher the pour point of the crude oil. Several researchers have concluded that the amount of chemical inhibitor required to mitigate wax deposition is a more prominent factor due to the interaction of the molecular structure between the wax molecules and the chemical inhibitor.^61−65^

In addition, WAT is the main factor affecting the growth and formation of solid wax molecules in the crude oil medium.^66^ Crude oil tends to be thicker below the WAT where the phases start to change from liquid to an amorphous solid phase. The degree of wax crystallization is now increased, which finally leads to the formation of more solid wax molecules.^53,67^ In the real field condition, an extremely cold temperature environment causes the temperature of the pipeline wall to drop below WAT and generate wax deposition. The higher the WAT, the higher the tendency for wax deposition to occur.

Extending the previous studies on the efficiency of 1% CPO and CPKO as wax inhibitors, this study focuses on confirming the highest performance of these palm oil inhibitors in mitigating wax deposition with 1% CPO and CPKO by using different samples of crude oil which is waxier.^40^ This paper reveals that palm oil inhibitors such as CPO and CPKO need different concentrations for different waxy crude oils in reaching the performance as wax inhibitors. Based on new findings, 1% CPO shows the highest performance as a wax inhibitor with 58.79% average PIE, followed by 1% CPKO with 54.75% efficiency. The average PIE of 1% CPO and 1% CPKO blend with Penara crude oil is lower compared to Mt Oversea Mckyle crude oil because Penara crude oil is waxier and contains a high WAT, a high pour point, and a high wax content. This indicates that palm oil inhibitors such as CPO and CPKO can inhibit wax deposition in waxy crude oil.

### 3.3 Effect on the Rheological of Crude Oil

**Figure 9:**
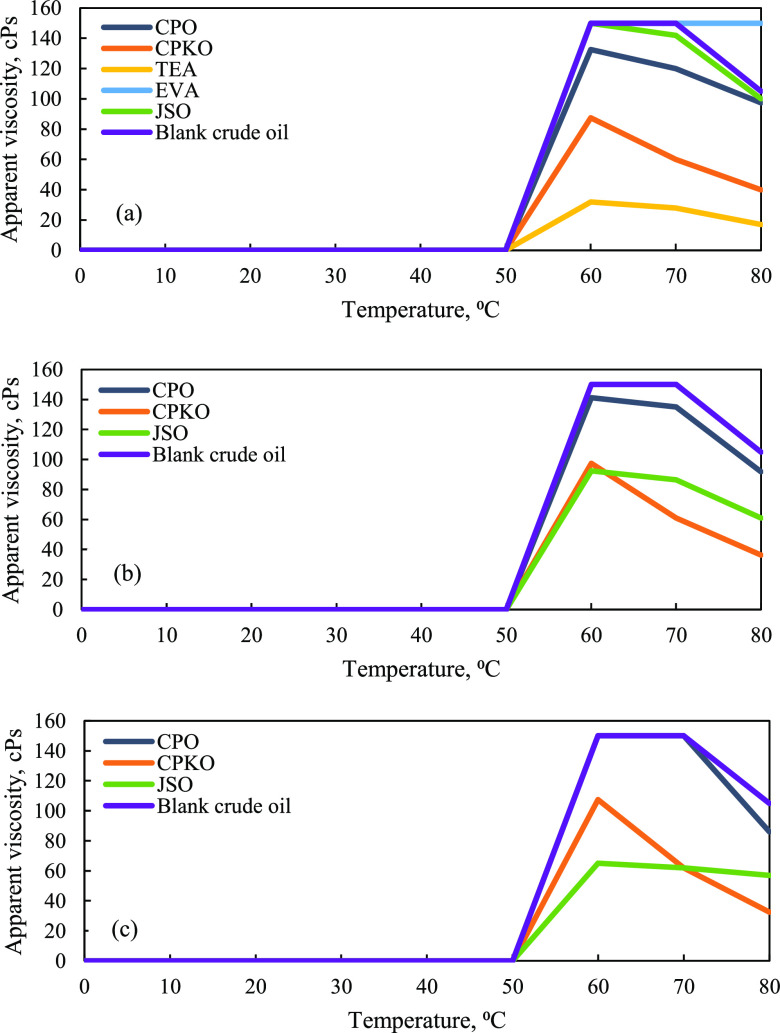
Apparent viscosity of crude oil versus temperature. (a) 1% concentration additives, (b) 3% concentration additives, and (c) 5% concentration additives.

The analysis of the wax deposition problem required viscosity study as a crucial aspect as its plays an important role in the flowability of crude oil. According to Ridzuan et al.,^68^ apparent viscosity is defined as the ratio between instantaneous shear stress and shear rate. Based on Figure 9a–c, all the natural plant-based additives, which are CPO, CPKO, and Malaysian JSO, were capable of reducing the apparent viscosity as compared to blank crude oil. The apparent viscosity of three natural plant-based additives proves that the apparent viscosity increases from 50 to 60 °C but decreases from 60 to 80 °C at 1, 3 and 5% concentrations. However, the apparent viscosity of crude oil with and without the presence of additives was constant at 0.00 cPs from 0 to 50 °C, as shown in Figure 9a–c.

The trend of 1% concentration at 60 °C (see Figure 9a) clearly shows that the apparent viscosity of CPO, CPKO, and Malaysian JSO was 132.50, 87.50, and 150.00 cPs, respectively. As the temperature increases from 60 to 80 °C, the trend of apparent viscosity for these three natural plant-based additives decreases to 97.50, 40.00, and 100.00 cPs, respectively. Even though 1% TEA shows the lowest apparent viscosity at temperatures from 50 to 80 °C, CPO, CPKO, and Malaysian JSO efficiently reduce the viscosity of Penara crude oil compared to other synthetic chemical inhibitors such as EVA at 1% concentration. This proves that these three natural plant-based additives can perform efficiently as flow improvers with a lower cost and are environmentally friendly compared to synthetic chemical inhibitors.

Based on Figure 9b, the trend of apparent viscosity for 3% concentration of the natural plant-based additive blend with Penara crude oil also decreases from 60 to 80 °C. At 60 °C, the apparent viscosity of CPO, CPKO, and Malaysian JSO was 141.25, 97.50, and 92.50 cPs, respectively. The value continuously decreases up to 91.75, 36.25, and 61.00 cPs, respectively, at 80 °C. A similar trend is found in Figure 9c, where these three natural plant-based additives decrease the apparent viscosity at 5% concentration with increasing temperature from 60 to 80 °C. At 60 °C, the apparent viscosity value of CPO, CPKO, and Malaysian JSO was 150.00, 107.50, and 65.00 cPs, respectively. The value decreases up to 86.00, 32.50, and 57.00 cPs, respectively, at 80 °C. Ultimately, this study reveals that 5% JSO blend with Penara crude oil gives the lowest apparent viscosity at 60 °C to 70 °C. Further, 5% CPKO shows the lowest apparent viscosity at 80 °C. This is a signal that the edible oils such as palm oil inhibitors (CPO and CPKO) and non-edible oils such as Malaysian JSO could interact with the paraffin wax molecules of Malaysia crude oil samples from 1 up to 5% concentration, thus reducing the viscosity of the samples.

Since the pour point temperature of Malaysia waxy crude oil used in this study is 59.25 °C, the temperature above this pour point value is considered as the boundary temperature which contributes to the flowing of normal crude oil. The solid wax formation dispersed in crude oil relates closely with WAT.^66^ Crude oil will change the phase from liquid to an amorphous solid below the WAT and tend to be thicker. The WAT of Penara crude oil from Malaysia basin used in this study is 72.24 °C. The increase in the degree of crystallization wax may cause a high formation of wax when the temperature is lower than WAT. This concludes that the amount of wax solid increases when the temperature decreases.^67^ Therefore, due to the high value of WAT and the pour point of Penara crude oil, the apparent viscosity of the Penara crude oil blend with and without inhibitors is constant at 0.00 cPs from 0 to 50 °C and the texture of samples is semi-solid.

According to Shigemoto et al.,^69^ wax molecules have more fast movement when the temperature is increased, which reduces the interaction tendency between the molecules. Therefore, the crude oil will possibly behave similar to a Newtonian fluid. However, the crude oil will become more viscous when the fluid starts to deviate from Newtonian to non-Newtonian behavior, and this situation occurs as soon as paraffin wax begins to precipitate in crude oil. The viscosity magnitude for a non-Newtonian fluid is varied depending on the force or applied stress. Meanwhile, a Newtonian fluid is a fluid whose viscosity does not depend on time or shear rate. Thus, to ensure the smooth transportation of crude oil in the pipeline system, more forces are needed to be applied. Based on the rheology method, the order of performances of natural plant-based additives used in this study which act as wax inhibitors at 60 °C (below WAT) is as follows:

5% JSO > 1% CPKO > 3% JSO > 3% CPKO > 5% CPKO > 1% CPO > 3% CPO > 1% JSO > 5% CPO.

This study concludes that the tendency of agglomeration and crystallization of wax is also connected to viscosity. The crystallization of solid wax occurred when the concentration of wax became closer to reach the supersaturation concentration, which reflects more viscous crude oil in the pipeline. A study of mitigation paraffin wax which leads to reduction of viscosity was successfully conducted with the addition of the wax inhibitors. Thus, according to Yi and Zhang,^70^ the functional group in the chemical inhibitor mostly controlled the solubility of the wax solid. From this study, it is confirmed that the use of palm oil inhibitors and Malaysian JSO with a fatty acid component such as oleic acid is the main factor contributing to the reduction of crude oil viscosity.^44,45^ From the Fourier transform infrared spectroscopy (FTIR) analysis conducted by Jayagobi et al.,^45^ C=O stretching carboxyl with the ester fatty acid group was found in Malaysian JSO, which proves the presence of oleic acid as the main component of this natural plant-based additive in improving the flowability of crude oil.

Therefore, the type of functional group in the selection of chemical inhibitors is important to control the solubility of asphaltene and paraffin wax molecules.^53^ According to Wang et al.,^71^ gelling of waxy crude oil that restricts the flow is caused by the deposition of wax at and below WAT. This condition produces obvious non-Newtonian behavior, which leads to the increment of apparent viscosity when the temperature of waxy crude oil achieves its pour point. In the worst scenario, higher deposition of paraffin wax molecules can be occurred, causing constricted flow and high frictional pressure losses. However, this study proves that natural plant-based additives such as CPO, CPKO, and Malaysian JSO are capable of decreasing the apparent viscosity at 60 and 70 °C of Penara crude oil below the WAT of crude oil.

**Figure 10:**
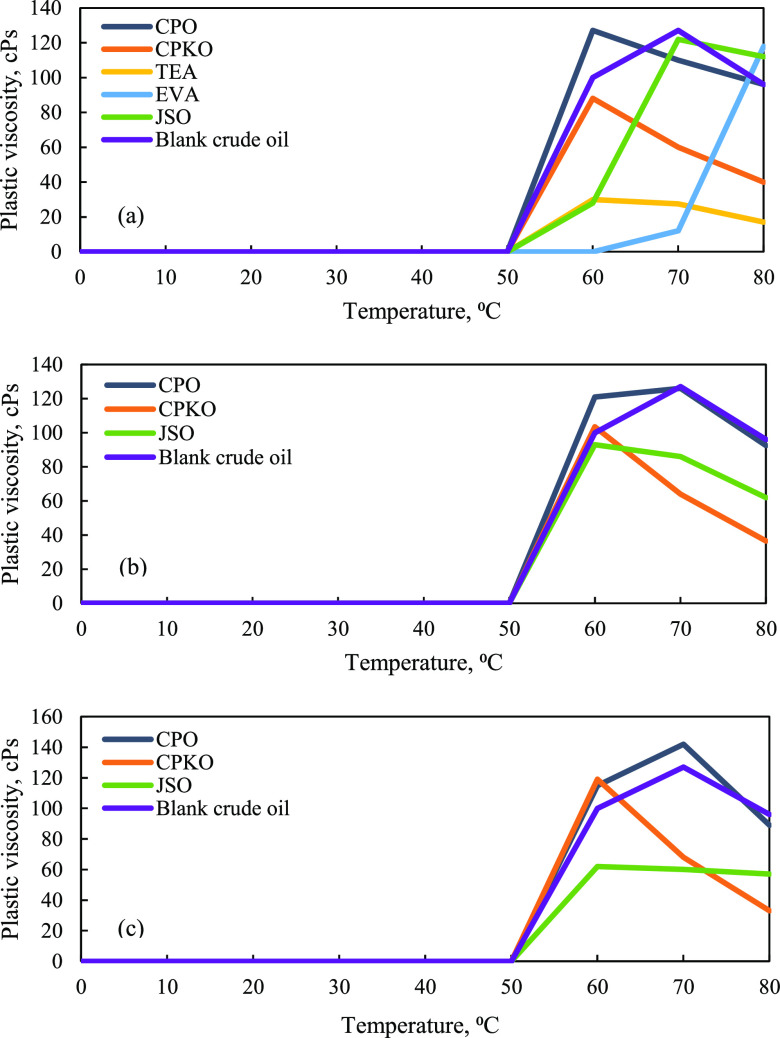
Plastic viscosity of crude oil versus temperature. (a) 1% concentration additives, (b) 3% concentration additives, and (c) 5% concentration additives.

In addition, Patel et al.^28^ mentioned that plastic viscosity is defined as the resistance of the fluid to flow. Based on Figure 10a–c, additives CPO, CPKO, and Malaysian JSO were found to decrease plastic viscosity to a significantly lower value as compared to the blank waxy crude oil at a certain concentration and temperature. The plastic viscosity for blank crude oil increases from 50 °C and starts to decline after 70 °C; meanwhile, the plastic viscosity with the presence of CPO, CPKO, and Malaysian JSO increases at 50 °C but starts to decrease after either 60 or 70 °C. Crude oil samples without and with additives at all concentrations show a constant value of plastic viscosity with 0.00 cPs from 0 to 50 °C.

Figure 10a–c shows the plastic viscosity of Penara crude oil without additives at 0 to 80 °C. The plastic viscosity of blank crude oil at 60 °C is 100.00 cPs and increases to 127.00 cPs at 70 °C. Then, the plastic viscosity starts to decrease at 80 °C with 96.00 cPs. According to Figure 10a, addition of 1% CPO in Penara crude oil increases the plastic viscosity up to 127.00 cPs at 60 °C compared to blank crude oil with 100.00 cPs. However, the plastic viscosity with addition of 1% CPKO and 1% JSO at 60 °C only increases to 88.00 and 28.00 cPs, respectively, which is lower than that of the blank crude oil. At 70 °C, addition of 1% CPO and 1% CPKO decreases the plastic viscosity to 110.00 and 60.00 cPs, respectively. However, 1% JSO increases the plastic viscosity up to 122.00 cPs at the same temperature, which is 70 °C. The trend of plastic viscosity decreases to 96.00 cPs (CPO), 40.00 cPs (CPKO), and 112.00 cPs (JSO) for these three natural plant-based additives with 1% concentration at 80 °C. Compared to synthetic chemical inhibitors, 1% TEA shows a great performance in reducing plastic viscosity at temperatures of 60, 70, and 80 °C. However, addition of 1% EVA increases the plastic viscosity of Penara crude oil from 60 to 80 °C, and it is found that natural plant-based additives perform efficiently in reducing the plastic viscosity compared to this synthetic chemical inhibitor.

At 3 and 5% concentrations of additives, the plastic viscosity trend is mimicking the previous apparent viscosity behavior. Generally, the trend increases from 50 to 60 °C and decreases from 60 to 80 °C. In this study, Malaysian JSO at 5% concentration could act as the best viscosity-reducing agent, followed by 1% CPKO and 3% JSO, due to their excellent performance in decreasing the plastic viscosity at 60, 70, and 80 °C. The results show that these natural plant-based additives not only prevent aggregation of waxy crystals but also destroy the crystalline structure of wax in Malaysia waxy crude oil. In addition, these viscosity-reducing agents also generate a dramatic change in the rheological behavior of the waxy crude oil. With an increased concentration of JSO, it is found that the rheological behavior changes significantly, and the change is most significant in decreasing the viscosity of crude oil in the presence of 5% JSO. This implies that various degrees of damage occurred in the internal structure of waxy crude oil due to the viscosity-reducing agent.^72^

The flow assurance of crude oil produces wax precipitation and affects the wax deposition rate.^38^ Consequently, the oil is trapped, and the amount of precipitation wax increases. This resulted in the increment of viscosity of crude oil, reduction of pressure loss, and the oil flow assurance in pipelines.^73^ The WAT of the blank crude oil in this study is 72.24 °C. When the temperature of crude oil falls below WAT, the waxes will form as plate-like crystals and bind together to generate a three-dimensional network. The rheological behavior of crude oil below WAT is commonly non-Newtonian. This behavior reinforces the fact mentioned by Majhi et al.^74^ that the kinematic viscosity of crude oil increases below WAT. The crude oil appears to be thicker below WAT as the molecules of crude oil change from the liquid phase to the amorphous solid phase.

However, in this study, it is found that addition of 5% JSO, 1% CPKO, and 3% JSO is able to reduce the viscosity of Penara crude oil at 60 and 70 °C, which are below the WAT. These additives successfully modify the wax molecules and delay the formation of wax crystals. As the temperature increases up to 80 °C, these natural plant-based additives continuously decrease the plastic viscosity of Penara crude oil. According to Gudala et al.^75^ and Kumar et al.,^76^ the alteration in rheological properties of crude oil was stipulated by decreasing viscosity due to the increase of temperature. Since the Penara crude oil has a high pour point temperature, which is 59.25 °C, the crude oil becomes immobile and solid wax formation occurs below this temperature. Based on Figure 10, there is no plastic viscosity for Penara crude oil with and without the presence of additives at 0 to 50 °C since the texture of crude oil was totally solid in this range of temperatures.

**Figure 11:**
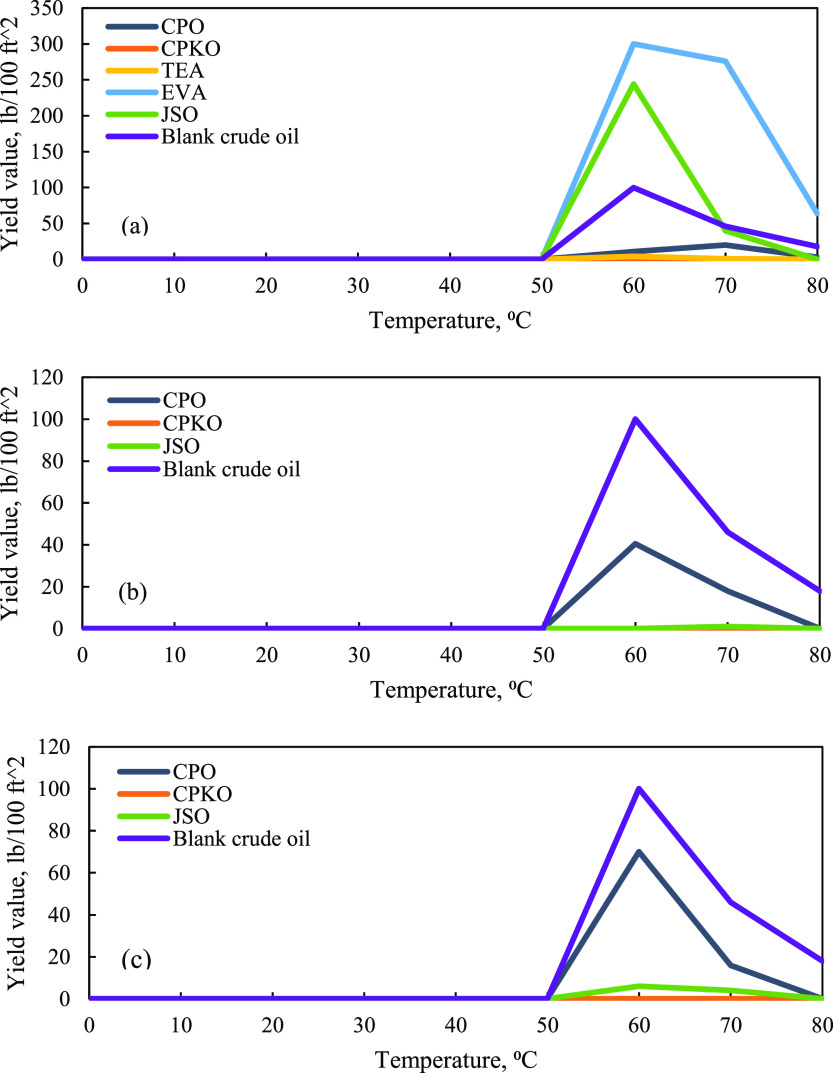
Yield value of crude oil versus temperature. (a) 1% concentration additives, (b) 3% concentration additives, and (c) 5% concentration additives.

Moreover, based on Patel et al.,^28^ yield value is defined as the resistance or stress required for a fluid to start flowing. Figure 11 shows the yield value of Penara crude oil without and with additives at 1, 3, and 5% concentrations versus temperature. Based on Figure 11a–c, the yield value of the waxy crude oil blend with natural plant-based additives such as CPO, CPKO, and Malaysian JSO is lower at a certain concentration and temperature compared with the yield value obtained from blank crude oil. However, all samples give an identical yield value at 0 to 50 °C with 0.00 lb/100 ft^2^.

Based on the graph in Figure 11a–c, the yield value of Penara crude oil without additives increases to 100.00 lb/100 ft^2^ at 60 °C. The yield value of this blank crude oil continuously decreases to 46.00 lb/100 ft^2^ at 70 °C and 18.00 lb/100 ft^2^ at 80 °C. Figure 11a shows that 1% CPO increases the yield value to 11.00 lb/100 ft^2^ at 60 °C and 20.00 lb/100 ft^2^ at 70 °C; then the yield value decreases to 3.00 lb/100 ft^2^ at 80 °C. However, 1% CPKO gives 0.00 lb/100 ft^2^ at 60 to 80 °C. Meanwhile, the yield value of 1% JSO increases up to 244.00 lb/100 ft^2^ at 60 °C, and this value is higher than that of the blank crude oil at the same temperature. At 70 °C, the yield value of 1% JSO decreases to 40.00 lb/100 ft^2^ and the value continuously decreases up to 0.00 lb/100 ft^2^ at 80 °C. Compared to synthetic chemical inhibitors, 1% TEA gives the yield value of 4.00 lb/100 ft^2^ at 60 °C, which then decreases to 1.00 lb/100 ft^2^ at 70 °C and 0.00 lb/100 ft^2^ at 80 °C. Meanwhile, 1% EVA increases the yield value of Penara crude oil up to 300.00 lb/100 ft^2^ at 60 °C, which then decreases to 276.00 lb/100 ft^2^ at 70 °C and 64.00 lb/100 ft^2^ at 80 °C. These findings prove that natural plant-based additives such as CPKO with 1% concentration perform efficiently compared to synthetic chemical inhibitors with the lowest yield value of 0.00 lb/100 ft^2^ at 60, 70, and 80 °C.

According to Figure 11b, the yield value of 3% CPO increases to 40.50 lb/100 ft^2^, but the value is still lower compared to the yield value of blank crude oil at 60 °C. Then, the yield value of 3% CPO continuously decreases to 18.00 lb/100 ft^2^ at 70 °C and 0.00 lb/100 ft^2^ at 80 °C. 3% CPKO gives a similar yield value from 60 to 80 °C with 0.00 lb/100 ft^2^, which is lower compared to the blank crude oil in the same range of temperatures. Meanwhile, 3% JSO gives 0.00 lb/100 ft^2^ at 60 °C, which then slightly increases to 1.00 lb/100 ft^2^ at 70 °C and decreases again to 0.00 lb/100 ft^2^ at 80 °C.

The trend in Figure 11c shows that yield value of 5% CPO increases to 70.00 lb/100 ft^2^ at 60 °C and then continuously decreases to 16.00 lb/100 ft^2^ at 70 °C and 0.00 lb/100 ft^2^ at 80 °C. 5% CPKO gives a similar yield value with 0.00 lb/100 ft^2^ at 60 to 80 °C. Meanwhile, the yield value of 5% JSO at 60 °C increases slightly to 6.00 lb/100 ft^2^; then the yield value continuously decreases to 4.00 lb/100 ft^2^ at 70 °C and 0.00 lb/100 ft^2^ at 80 °C. However, all the yield values of these three natural plant-based additives at 5% concentration are lower compared to the yield value of blank crude oil in the temperature range of 60 to 80 °C.

Based on the observation, yield values in this study clearly showed that with an increase in the temperature from 60 to 80 °C, a significant decrease in yield values was observed. From this study, it is found that CPKO at all concentrations (1, 3, and 5%) gives the lowest yield value from 60 to 80 °C, followed by 3% JSO and 5% JSO. The performance of these natural plant-based additives is better compared to synthetic chemical inhibitors such as TEA and EVA. A lower yield value will lead to the easy flow of crude oil inside the pipeline due to less stress required for the fluid to start flowing. Therefore, the power expected to run the pump for crude oil transportation will be low. This condition will contribute to energy sustainability and give a good impact on crude oil production. This proved that natural plant-based additives such as CPO, CPKO, and Malaysian JSO are suitable as natural wax inhibitors in mitigating wax deposition and improve the rheology of Penara waxy crude oil from Malaysia basin.

In addition, the gel strength was studied as a function of shear, thermal histories, and mixture composition during gelation. Gel strength study is vital in restarting flow in a gelled pipeline.^77^ The gel strength value of Penara waxy crude oil was obtained from reading at a shear rate of 3 rpm using a Fann viscometer. Table 4 shows the gel strength of crude oil with and without additives. The reading was taken at temperatures of 60, 70, and 80 °C. Based on Table 4, the gel strength of blank crude oil without the presence of additives is 7.00 lb/100 ft^2^ at 60 °C, 5.00 lb/100 ft^2^ at 70 °C, and 4.00 lb/100 ft^2^ at 80 °C. With the addition of natural plant-based additives, it is found that the gel strength of crude oil decreases at temperatures of 60, 70, and 80 °C compared to the gel strength of blank crude oil.

**Table 4:** Gel Strength of Penara Crude Oil with and without Additives

		gel strength, lb/100 ft2		
type of sample	concentration of the additive, %	60 °C	70 °C	80 °C
crude oil		7.00	5.00	4.00
CPO	1	4.00	3.50	3.50
	3	4.25	3.75	3.25
	5	4.50	4.00	3.00
CPKO	1	2.50	2.00	2.50
	3	2.75	2.50	2.25
	5	3.00	3.00	2.00
JSO	1	5.50	4.50	3.00
	3	4.00	3.00	3.00
	5	4.00	4.50	4.00
TEA	1	2.50	2.00	2.00
EVA	1	6.50	9.00	11.00

According to Table 4, the gel strengths of 1% concentration of CPO, CPKO, and Malaysian JSO at 60 °C were 4.00 lb/100 ft^2^, 2.50 lb/100 ft^2^, and 5.50 lb/100 ft^2^, respectively. At 70 °C, the gel strength of 1% CPO decreases to 3.50 lb/100 ft^2^ and the gel strength value is constant up to 80 °C. The gel strength of 1% CPKO decreases to 2.00 lb/100 ft^2^ at 70 °C and then increases to 2.50 lb/100 ft^2^ at 80 °C. Meanwhile, the gel strength of 1% JSO continuously decreases to 4.50 lb/100 ft^2^ at 70 °C and 3.00 lb/100 ft^2^ at 80 °C. Compared to synthetic chemical inhibitors, the gel strength of TEA is low with 2.50 lb/100 ft^2^ at 60 °C and 2.00 lb/100 ft^2^ at 70 and 80 °C. From Table 4, it can be clearly seen that 1% CPKO can act efficiently as a synthetic chemical inhibitor such as TEA, which is well known as a wax inhibitor. Meanwhile, 1% EVA gives the highest gel strength as compared to blank crude oil and other natural plant-based inhibitors with 6.50 lb/100 ft^2^ at 60 °C, 9.00 lb/100 ft^2^ at 70 °C, and 11.00 lb/100 ft^2^ at 80 °C.

Table 4 shows the gel strength of 3% CPO with 4.25 lb/100 ft^2^ at 60 °C, which then continuously decreases to 3.75 lb/100 ft^2^ at 70 °C and 3.25 lb/100 ft^2^ at 80 °C. Addition of 3% CPKO in Penara crude oil gives 2.75 lb/100 ft^2^ gel strength value at 60 °C; then the value decreases to 2.50 lb/100 ft^2^ at 70 °C and 2.25 lb/100 ft^2^ at 80 °C. Meanwhile, the gel strength of 3% JSO is 4.00 lb/100 ft^2^ at 60 °C; then the value decreases to 3.00 lb/100 ft^2^ at 70 °C and becomes constant up to 80 °C. With 5% concentration of CPO, the gel strength of Penara crude oil at 60 °C is 4.50 lb/100 ft^2^, which then decreases to 4.00 lb/100 ft^2^ at 70 °C and 3.00 lb/100 ft^2^ at 80 °C. Meanwhile, the gel strength of 5% CPKO is constant at 60 and 70 °C with 3.00 lb/100 ft^2^; then the value decreases to 2.00 lb/100 ft^2^ at 80 °C. Last, the gel strength of 5% JSO is 4.00 lb/100 ft^2^ at 60 °C, which then slightly increases to 4.50 lb/100 ft^2^ at 70 °C and finally decreases again to 4.00 lb/100 ft^2^ at 80 °C.

Based on the findings obtained in Table 4, it is found that the crude oil blend with 1% CPKO has the lowest gel strength at 60 and 70 °C, which is below the WAT of Penara crude oil. 1% CPKO shows its capability as a natural plant-based additive when the performance of gel strength is similar with commercial synthetic wax inhibitors such as TEA. Moreover, CPO, CPKO, and Malaysian JSO at all concentrations (1, 3, and 5%) give a great performance in reducing gel strength and perform better compared to other synthetic chemical inhibitors such as EVA at 60 to 80 °C. This proves that CPO, CPKO, and Malaysian JSO have high potential as natural plant-based additives to solve flow assurance problems in Penara oilfield Malaysia basin.

A waxy crude oil which gels below WAT has been investigated under dynamic conditions using the rheological method. According to Visintin et al.,^36^ the ultimate strength of the waxy crude oil gel can be reduced below the pour point temperature. A sharp transition in gel strength at the pour point temperature occurred when n-paraffins dissolved in organic solvents. However, the values of gel strength discussed in this study were determined at a temperature of 60 °C and above. This temperature is close to the pour point temperature of Penara waxy crude oil, which is 59.25 °C. Below this pour point temperature, the crude oil starts to immobilize and finally turns to solid with the decrease of temperature, and the rheological test cannot be conducted. Therefore, the natural plant-based additives with 1, 3, and 5% concentrations used at 60, 70, and 80 °C were easily soluble in waxy crude oil and retarded the growth of wax crystals, resulting in lower gel strength and smaller crystals.

## 4 Conclusions and Recommendation

The ability of natural plant-based additives as wax inhibitors and flow improvers on waxy crude oil were examined in this study. It can be concluded that CPO and CPKO obtained from Malaysia’s oil palm plantation and JSO from the Malaysian origin could be used as wax inhibitors for Penara waxy crude oil of Malaysia basin which is waxier compared to Mt Oversea Mckyle Arab heavy crude oil and Chenor crude oil. Natural CPO, CPKO, and Malaysian JSO perform efficiently in reducing the amount of wax deposited and decrease the viscosity of waxy crude oil compared to the uses of commercial synthetic chemical inhibitors such as EVA. The active component of both palm oil inhibitors and Malaysian JSO, which were involved with the higher hydrocarbon in the crude oil, is oleic acid. These interactions contribute to a high average of PIE with 86.30% of 5% JSO, followed by 85.49% of 3% JSO, 58.79% of 1% CPO, and 54.75% of 1% CPKO, when these natural plant-based additives were added to the Penara crude oil sample. CPO, CPKO, and Malaysian JSO effectively reduced the apparent viscosity, plastic viscosity, yield value, and gel strength of Penara waxy crude oil to significantly lower values. At a temperature of 60 °C below WAT, 5% JSO and 1% CPKO were acting as the highest viscosity-reducing agents. These additives can be effectively used as viscosity index improvers. The usage of natural plant-based additives efficiently reduces the quantity of wax as a waste for sustainable production and prevents contaminant groundwater due to spillage of commercial synthetic chemical inhibitors. Further research is suggested utilizing these natural plant-based additive blends with several types of waxy crude oils under dynamic flow conditions, rheological behavior with different equipment such as rheometers and viscometry, pour point, WAT measurements using DSC, and wax morphology using scanning electron microscopy or optical light microscopy. Besides this, characterization of crude oil using saturate, aromatic, resin, and asphaltene analysis is also recommended for future studies.

## NOMENCLATURE

API: American Petroleum Institute
CPKO: crude palm kernel oil
CPO: crude palm oil
CSO: castor seed oil
DSC: differential scanning calorimetry
EVA: ethylene-*co*-vinyl-acetate
FTIR: Fourier transform infrared spectroscopy
GCMS: gas chromatography mass spectrometry
JSO: jatropha seed oil
PIE: paraffin inhibition efficiency
RSO: rubber seed oil
SEM: scanning electron microscopy
TEA: triethanolamine
WAT: wax appearance temperature
